# Supplementary material for: Anthropogenic Disturbances Influenced the Island Effect on Both Taxonomic and Phylogenetic Diversity on Subtropical Islands, Pingtan, China
Source: Plants (Basel). 2024 Jun 1;13(11):1537. doi: 10.3390/plants13111537 (PMC11174741; doi:10.3390/plants13111537)
Supplement: Supplementary file 1 [file plants-13-01537-s001.zip › plants-3031572-supplementary.pdf]

Supplementary files

Figure S1 Explanation percentage of variance for different combinations of PCA one and two axes. G1, Island effect (island area + isolation). G2, Island spatial heterogeneity. G3, Anthropogenic disturbances G12, PCA value of island effect + island spatial heterogeneity. G13, PCA value of island effect + anthropogenic disturbances. G23, PCA value of island spatial heterogeneity + human-induced disturbance. G123, PCA value of island effect + island spatial heterogeneity + anthropogenic disturbances. A, Island area effect. B, Island isolation effect. C, Coastline length. D, Elevation E, Nearest island distance. F,Shape index. G, Distance to Haitan island. H,Land use.

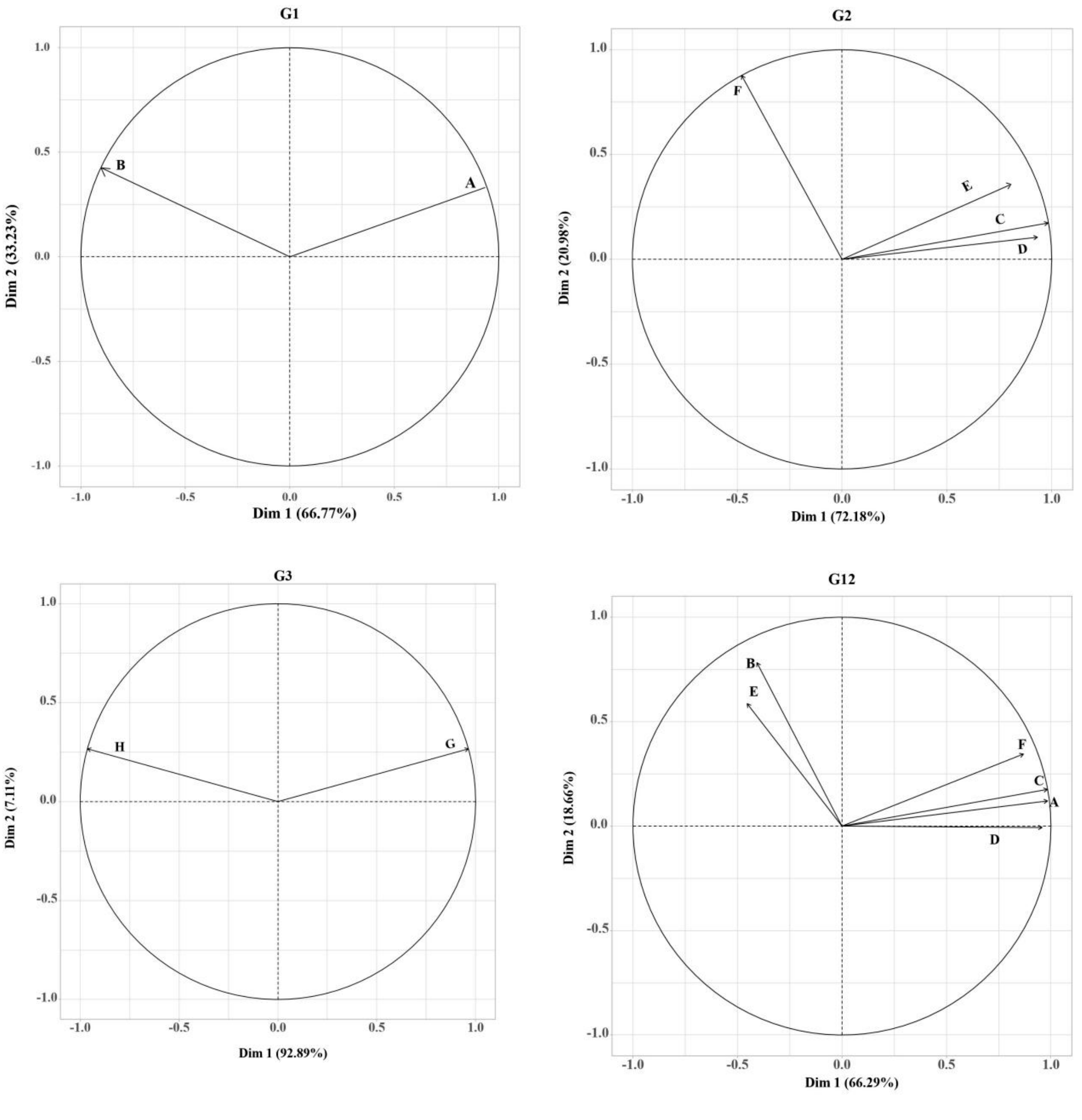

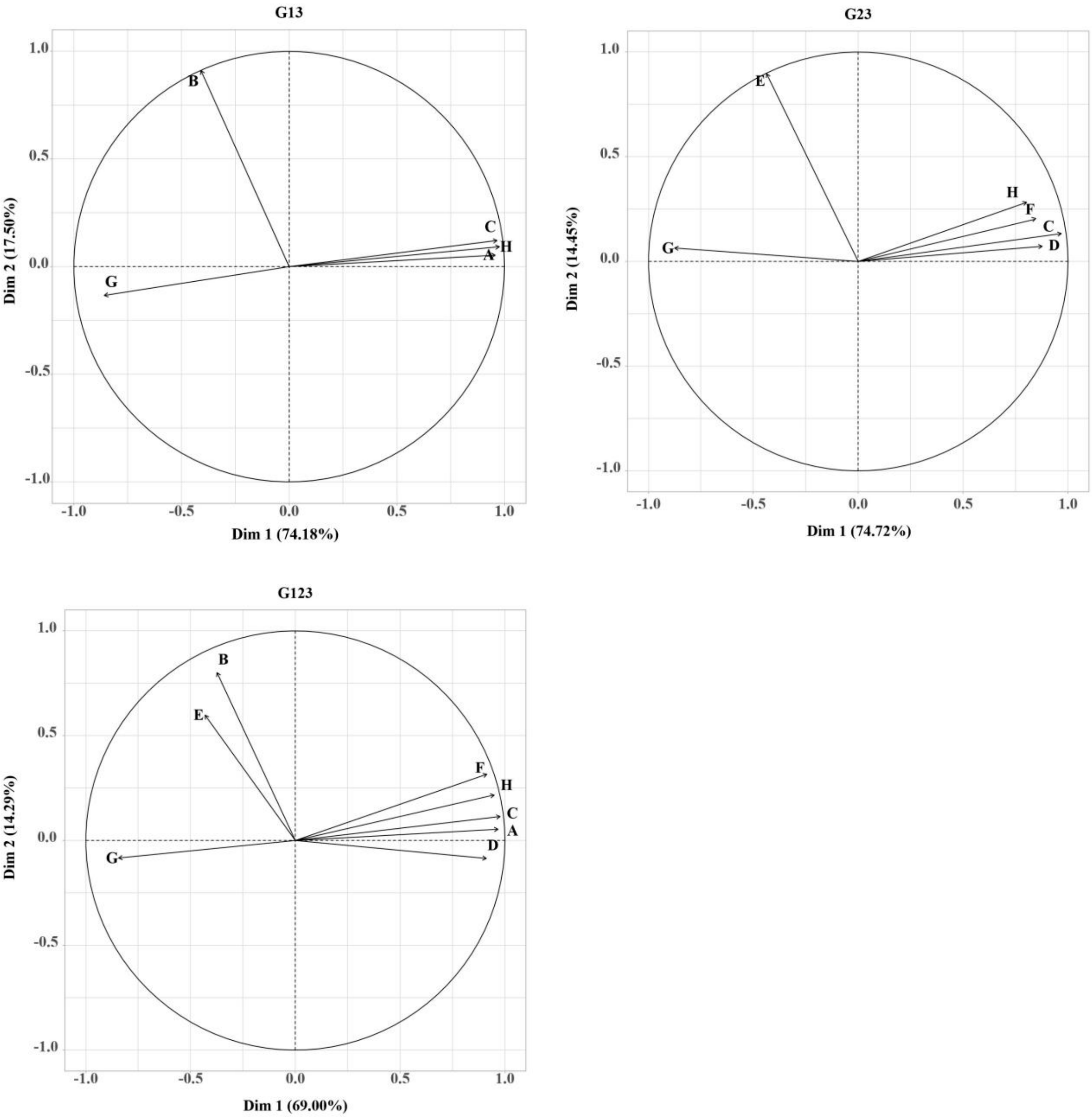

Figure S2 The linear relationship between island area and phylogenetic structure, where *SES.MPD* stands for Standardized Effect Size of Mean Pairwise Distance, and *SES.MNTD* stands for Standardized Effect Size of Mean Nearest Taxon Distance. The dashed line represents the best-fitting linear regression line, and the gray area indicates the 95% confidence interval around the line.

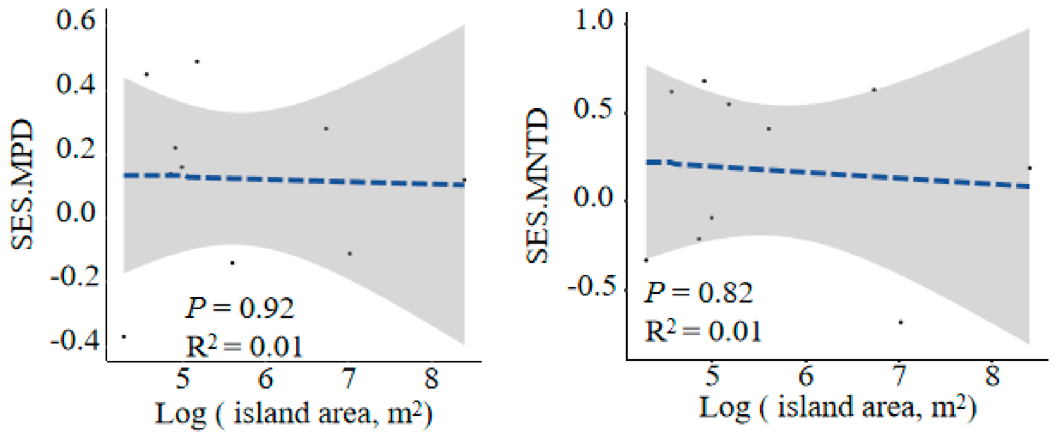

Figure S3 The linear relationship between isolation and taxonomic and phylogenetic diversity, and structure, where MD stands for Distance to the mainland, SR, Species Richness. SES.PD, Standardized Effect Size of Phylogenetic Diversity. *ES.MPD*, Standardized Effect Size of Mean Pairwise Distance. and *SES.MNTD*, Standardized Effect Size of Mean Nearest Taxon Distance. The blue line and dashed line represent the best-fitting linear regression lines. and the gray area indicates the 95% confidence interval around the lines.

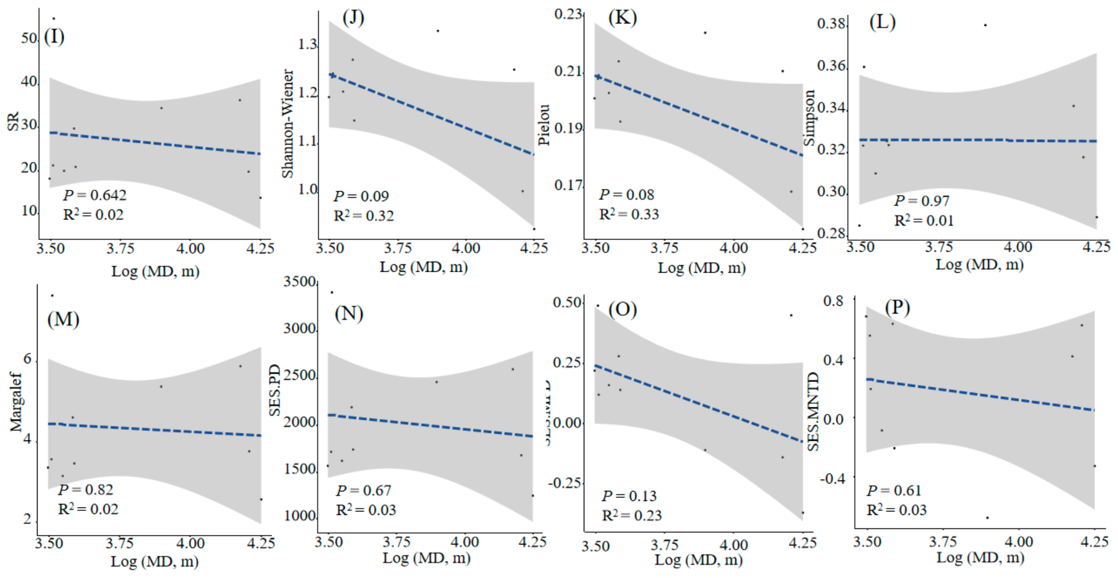

Figure S4 The correlation analysis between vegetation community taxonomic and phylogenetic indices on Pingtan Islands. Red indicates a positive correlation, blue indicates a negative correlation. The darker the color and the larger the circle, the stronger the correlation. SR, Species Richness. SES.PD, Standardized Effect Size of Phylogenetic Diversity. *NRI*, Net Relatedness Index. *NTI*, Net Nearest Taxon Index. *, $P < 0.05$, **, $P < 0.01$, ***, $P < 0.001$.

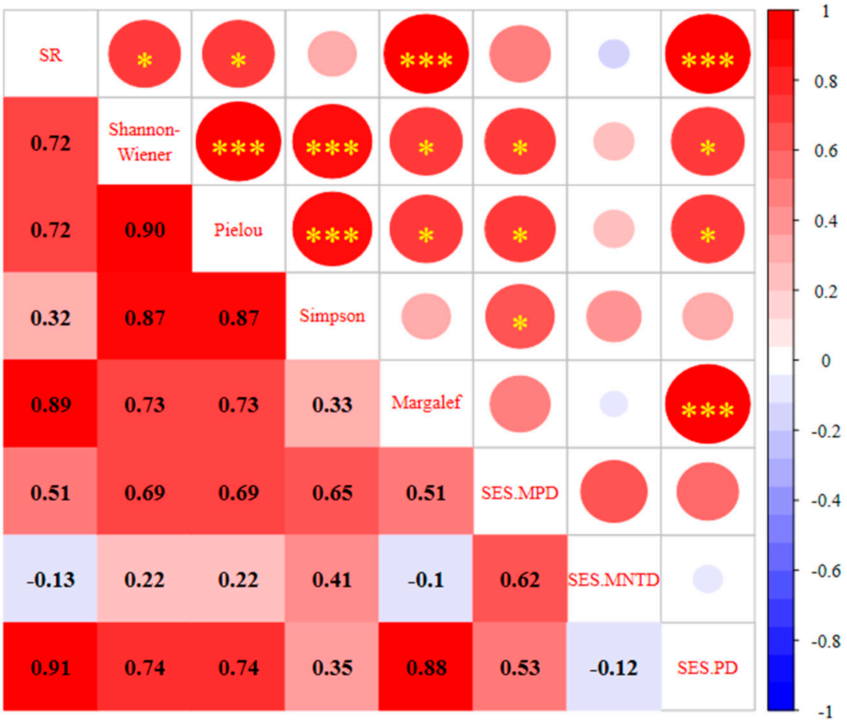

Table S1 Summary of basic information of the investigated islands

Number of sample plots	Island name	Longitude	Latitude	island area (m2)	coastline length (m)	distance to the mainland (m)	Elevation (m)	Nearest Island Distance (m)	Shape Index	Distance to the Hantan Island (m)	Land use (%)
18	Hantan Island	119° 47′ 46″	25° 31′ 28″	250124271.58	216938.68	3249.63	431.00	292.70	3.37	0.00	74.12
9	Dalian Island	119° 40′ 36″	25° 38′ 32″	10440210.28	20254.04	7880.23	227.00	616.60	1.54	7850.00	15.41
9	Cao Island	119° 43′ 29″	25° 23′ 31″	5400103.21	16088.25	3839.74	207.00	470.60	1.70	3970.00	24.17
6	Jiangshan Island	119° 48′ 18″	25° 26′ 31″	402418.37	4722.98	15008.67	59.00	335.30	1.83	1010.00	1.11
6	Beiguan	119° 39′ 16″	25° 22′ 55″	149868.69	1739.46	3228.31	42.00	850.00	1.10	3200.00	0.24

	Island										
9	Monkey Island	119° 40′ 10″	25° 29′ 51″	98187.31	1245.33	3537.73	53.00	247.50	0.98	2800.00	0.10
3	Nanguan Island	119° 40′ 55″	25° 20' 40″	81666.26	1169.42	3141.58	40.00	805.40	1.01	7500.00	0.13
6	Huang Island	119° 40′ 50″	25° 27′ 36″	71845.16	1417.30	3888.86	26.00	255.80	1.30	2700.00	0.22
3	Baijiang Island	119° 48′ 54″	25° 25′ 30″	36656.84	1092.23	16121.72	18.00	1674.00	1.40	3900.00	1.41
3	Qing Island	119° 48′ 41″	25° 31′ 8″	19467.41	620.64	17768.69	14.00	558.20	1.09	5200.00	0.16

Table S2 Taxonomic and phylogenetic diversity of different islands in Pingtan. SR, Species richness. SES.PD, standardization phylogenetic diversity. *SES.MPD*, Standardized Effect Size of Mean Pairwise Distance. *SES.MNTD*, Standardized Effect Size of Mean Nearest Taxon Distance. S-W, Shannon-Wiener index.

Island name	SR	SES.PD	*SES.MPD*	*SES.MNTD*	S-W	Pielou	Simpson	Margalef
Hantan Island	55.29	3417.33	0.12	0.19	1.20	0.20	0.29	7.65
Dalian Island	34.53	2460.35	-0.11	-0.68	1.21	0.20	0.31	5.38
Cao Island	29.79	2191.08	0.28	0.63	1.24	0.21	0.32	4.61
Jiangshan Island	36.36	2597.03	-0.14	0.41	1.27	0.21	0.33	5.89
Beiguan Island	21.24	1715.71	0.49	0.55	1.25	0.21	0.34	3.57
Monkey Island	19.97	1618.97	0.16	-0.09	1.15	0.19	0.32	3.15
Nanguan Island	18.17	1565.82	0.22	0.68	1.33	0.22	0.38	3.36
Huang Island	20.88	1739.83	0.14	-0.21	0.92	0.16	0.29	3.46
Baijiang Island	19.78	1678.43	0.45	0.62	1.25	0.21	0.36	3.76
Qing Island	13.73	1247.80	-0.37	-0.33	1.00	0.17	0.32	2.57

Table S3 Principal Component Analysis (PCA) scores for the first axis of island effect, island spatial heterogeneity, and anthropogenic disturbances. G1, Island effect (island area + isolation). G2, Island spatial heterogeneity. G3, Anthropogenic disturbances. G12, PCA value of island effect + island spatial heterogeneity. G13, PCA value of island effect + anthropogenic disturbances. G23, PCA value of island spatial heterogeneity + human-induced disturbance. G123, PCA value of island effect + island spatial heterogeneity + anthropogenic disturbances.

G1	G2	G3	G12	G23	G123
-0.26	-0.42	0.18	0.40	0.97	0.97
-0.29	0.68	0.74	-0.25	1.17	1.29
0.16	0.93	0.03	0.84	0.04	-0.10
1.21	1.03	-0.47	1.28	-0.66	-0.66
-0.25	0.01	0.34	0.50	0.70	0.70
-0.21	0.54	0.07	0.61	0.56	0.56
1.08	0.97	-0.33	0.89	-0.10	0.04
-0.30	-0.52	1.02	-0.43	1.29	1.17
-2.54	-4.53	1.38	-5.39	1.50	1.50
1.40	1.31	-2.95	1.54	-5.47	-5.47

Table S4 Linear analysis results of different combinations (island effects, island spatial heterogeneity, and anthropogenic disturbances) on taxonomic and phylogenetic diversity with no significant correlation. SR, Species Richness. SES.PD, Standardized Effect Size of Phylogenetic Diversity. *NRI*, Net Relatedness Index. G1, Island Effect (Island Area + Isolation). G2, Island Spatial Heterogeneity. G3, Anthropogenic disturbances. G12, PCA values of Island Effect + Island Spatial Heterogeneity. G13, PCA values of Island Effect + Anthropogenic disturbances. G23, PCA values of Island Spatial Heterogeneity + Anthropogenic disturbances. G123, PCA values of Island Effect + Island Spatial Heterogeneity + Anthropogenic disturbances.

Taxonomy diversity index	influence factor	R^2	*T*-value	*P*-value
SES.MPD	G1	0.318	2.125	0.081

	G2	0.376	2.251	0.059
	G3	0.288	-1.874	0.150
	G23	0.202	-1.366	0.186
	G123	0.241	-1.635	0.154
SES.MNTD	G1	0.013	0.154	0.954
	G2	0.065	0.498	0.621
	G3	0.102	-0.236	0.698
	G12	0.039	0.369	0.498
	G13	0.059	-0.695	0.478
	G23	0.031	-0.398	0.678
	G123	0.019	-0.395	0.656
SR	G2	0.338	2.023	0.078
	G12	0.294	1.817	0.078
	G13	0.397	-2.017	0.078
SES.PD	G2	0.387	2.646	0.055
	G12	0.386	2.241	0.055
	G13	0.386	-2.243	0.055
Shannon-Wiener	G23	0.380	-1.915	0.058
	G123	0.386	-2.444	0.055
Pielou	G23	0.380	-2.215	0.058
	G123	0.386	-2.244	0.055
Simpson	G1	0.314	1.914	0.092

	G3	0.121	-1.048	0.325
	G23	0.060	-0.712	0.497
	G123	0.065	-0.747	0.476
	G2	0.375	2.192	0.060
Margalef	G12	0.361	2.126	0.066
	G13	0.370	-2.167	0.062
